# Sex-specific associations between gut microbiota and myopia in adolescents: a clinical predictive modeling study

**DOI:** 10.3389/fcimb.2026.1778840

**Published:** 2026-05-28

**Authors:** Zichen Luan, Yucui Zheng, Ning Zhang, Yamin Chen, Shumeng Wei, Guiyu Ren, Fuming Kong, Dianhuan Ju, Rui Hao

**Affiliations:** 1 Clinical College of Ophthalmology, Tianjin Medical University, Tianjin, China; 2 Tianjin Eye Hospital, Tianjin Key Lab of Ophthalmology and Vision Science, Tianjin Eye Institute, Tianjin, China; 3 Department of Ophthalmology, Eco-city Hospital of Tianjin Fifth Central Hospital, Tianjin, China; 4 Nankai University Eye Institute, Nankai University Affiliated Tianjin Eye Hospital, Tianjin, China

**Keywords:** 16S rRNA sequencing, adolescents, gut microbiota, gut–eye axis, myopia, random forest model

## Abstract

**Introduction:**

Myopia is a common refractive disorder in adolescents, and its association with the gut microbiota remains incompletely defined.

**Methods:**

We performed 16S rRNA sequencing on fecal samples from 102 adolescents (49 myopic, 53 non-myopic; aged 6–18 years) and analyzed microbial diversity, taxonomy, function, and random forest-based classification, stratified by sex

**Results:**

Myopic adolescents showed lower gut microbial richness (observed and Chao1, both P < 0.05) than non-myopic controls, but overall community structure did not differ. Sex-stratified analyses revealed that these diversity reductions occurred only in myopic males (all α-diversity indices, P < 0.01), not in females. Several genera differed between myopic and non-myopic males (e.g., lower Akkermansia, Alistipes, Oscillibacter; higher Veillonella, Sutterella), whereas fewer differences were found in females. Functional predictions indicated altered metabolism and immune pathways. A random forest model achieved moderate overall accuracy (AUC = 76.84%), with higher performance in males (89.13%) than females (69.21%).

**Discussion:**

Adolescent myopia is associated with reduced gut microbial richness and sex-specific compositional changes, particularly in males, underscoring the importance of sex in gut–eye axis research.

## 1 Introduction

Myopia is a refractive error caused by light focusing in front of the retina, primarily characterized by a reduction in distance vision and impaired visual quality. High myopia is associated with an increased risk of sight-threatening ocular complications that may result in irreversible vision loss (Du et al., 2024; Khanal et al., 2025). Over recent decades, the global prevalence of myopia has risen steadily, rendering it a major public-health concern worldwide (Zhang et al., 2025). Although genetic predisposition and environmental exposure are widely recognized as principal risk factors for myopia, these factors alone do not fully explain the substantial inter-individual variability observed in disease onset and progression (Ying et al., 2024), emerging evidence suggests that systemic factors beyond ocular parameters may also contribute to pathophysiology of myopia (Omar et al., 2024; Sun et al., 2024).

The gut microbiota represents the largest and most complex microbial ecosystem in the human body and exerts profound effects on host immune regulation, metabolic homeostasis, and inflammatory responses (Onisiforou et al., 2025). With rapid progress in microbiome research, the gut–brain axis and, more recently, the gut–eye axis have gained increasing attention as prominent areas of study. Accumulating studies have shown that gut microbial dysbiosis is linked to various ophthalmic disorders, including diabetic retinopathy, glaucoma, keratitis, and age-related macular degeneration (Fu et al., 2023). Furthermore, several studies using 16S rRNA sequencing have reported associations between gut microbial composition and myopia; however, the proposed microbial signatures and their predictive performance remain inconsistent across studies (Li et al., 2024; Omar et al., 2024; Sun et al., 2024). Li et al. combined clinical analyses with animal experiments and demonstrated that gut microbial dysbiosis reduces circulating levels of the microbial metabolite indole-3-acetic acid (3-IAA), thereby attenuating scleral collagen synthesis via the SP1–COL1A1 pathway and promoting high-myopia progression. Restoration of a healthy microbiota or supplementation with 3-IAA significantly delayed this process (Li et al., 2024). In addition, Mendelian randomization analyses suggest that the gut microbiota may influence myopia development through modulation of host lipid metabolism (Hui et al., 2025).

In the present study, 16S rRNA sequencing was applied to fecal samples obtained from 102 adolescents to investigate associations between gut microbiota composition and myopia, with a particular focus on sex-specific differences. We further evaluated the predictive value of gut microbial features using a machine-learning approach.

## 2 Materials and methods

### 2.1 Participant recruitment and data collection

Between January and August 2025, a total of 102 participants aged 6–18 years were recruited from the ophthalmology outpatient clinic of the Eco-City Branch of Tianjin Fifth Central Hospital. The cohort comprised 49 adolescents with myopia and 53 non-myopic controls. All participants underwent comprehensive ophthalmic examinations, including slit-lamp evaluation of the anterior segment and detailed medical history assessment. Visual acuity was measured in all participants, and cycloplegic refraction was performed in those diagnosed with myopia. Based on refractive status and sex, participants were stratified into four subgroups,including 26 male myopic cases, 27 female myopic cases, 27 male non-myopic cases, and 22 female non-myopic cases.

Inclusion criteria were age between 6 and 18 years with corrected visual acuity of 0.00 logMAR, no use of antibiotics or probiotic gastrointestinal medications within the preceding three months, and no history of gastrointestinal surgery or organic gastrointestinal disease. Exclusion criteria included amblyopia or strabismus, recent use of contact lenses or orthokeratology lenses, recent topical ocular medication use, and systemic diseases known to affect ocular health.

The study adhered to the principles of the Declaration of Helsinki and was approved by the Medical Ethics Committee of Tianjin Fifth Central Hospital (Approval No.: Tianjin Medical Ethics Committee Review [2025] No. 14). Written informed consent was obtained from the legal guardians of all participants.For the sake of clarity, the subsequent analysis divides the participants into four subgroups: ME (non-myopic males), MM (myopic males), FE (non-myopic females), and FM (myopic females).

### 2.2 Fecal sample collection and processing

Participants were provided with sterile, single-use fecal collection kits containing DNA preservation solution. After voiding the bladder, stool samples were collected using disposable sampling paper placed over the toilet seat. Approximately a soybean-sized aliquot from the middle portion of the stool was transferred into the preservation solution, sealed immediately, and stored at −80 °C until analysis.

Genomic DNA was extracted using the TIANamp Stool DNA Kit according to the manufacturer’s instructions. The V4 hypervariable region of the bacterial 16S rRNA gene was amplified using universal primers:515F (5’-GTGCCAGCMGCCGCGGTAA-3’) and 806R (5’-GGACTACHVGGGTWTCTAAT-3’).

The PCR reaction mixture contained 10 ng of genomic DNA template, 0.2 μM of forward and reverse primers, and 15 μL of Phusion High-Fidelity PCR Master Mix. The amplification protocol consisted of an initial denaturation at 98 °C for 1 min; followed by 30 cycles of denaturation at 98 °C for 10 sec, annealing at 50 °C for 30 sec, and extension at 72 °C for 30 sec; with a final extension at 72 °C for 5 min.

PCR products were purified, pooled, and subjected to high-throughput sequencing following successful library construction.

### 2.3 Bioinformatics and microbiome analysis

Sequencing data were processed using QIIME2. Amplicon sequence variants were generated via DADA2 denoising and taxonomically annotated using the SILVA reference database.Alpha-diversity indices were calculated on normalized datasets, and beta-diversity was assessed using weighted UniFrac distances. Principal coordinate analysis was performed to visualize community-level differences, PCoA was conducted and visualized using the ade4 and ggplot2 packages in R software (version 4.0.3).

The distribution histograms of relative abundance were generated using the SVG function in Perl. Differences in taxonomic abundance between groups were calculated in R using the metagenomeSeq package. Linear discriminant analysis effect size (LEfSe) was applied to identify biomarkers with statistically significant differences among groups; analyses and visualizations were performed using the standalone LEfSe software, with the threshold set to an LDA score > 3. Functional profiles of the gut microbiota were predicted using Tax4Fun (version 0.3.1).

### 2.4 Statistical analysis

All statistical analyses were performed using R version 3.4.0. For microbial alpha diversity, the observed species index and the Chao1 index were used to assess community richness, while the Shannon and Simpson indices were used to evaluate community diversity. Differences in alpha and beta diversity indices between groups were tested using the Wilcoxon rank-sum test. Based on the KEGG database, Tax4Fun was employed to predict the biological functions of the gut microbiota associated with myopia, and differences in functional pathway abundances between groups were assessed via Student’s t-test. A P-value of < 0.05 was considered statistically significant.

## 3 Results

### 3.1 Clinical characteristics of participants

Researchers recruited 102 subjects at the ophthalmology outpatient clinic of Tianjin Fifth Central Hospital Eco-City Branch, including 53 myopic patients and 49 healthy controls (comprising emmetropic and mild hyperopic individuals).For these healthy controls, the spherical equivalent (SE) ranged from +2.00 D to -10.25 D, and the axial length (AL) ranged from 21.97 mm to 27.36 mm. The average age of participants was 11.35 ± 3.11 years, ranging from 6 to 18 years old. Questionnaire results (**Table 1**) revealed significant differences in the prevalence of picky eating and selective eating between the myopic and non-myopic groups (P = 0.033), in addition to genetic (parental visual acuity) and environmental factors (screen time). This suggests that adolescents’ dietary behaviors may be associated with their myopic status.

**Table 1:** Baseline data of participants.

General data	Emmetropia (n= 49)	Myopia (n= 53)	t/χ^2^	P
Age (mean ± SD, years)	10.88 ± 3.26	11.79 ± 2.96	-1.484	0.141
BMI	19.22 ± 4.28	19.72 ± 4.27	-0.58	0.565
SE	0.43 ± 0.43	-2.92 ± 2.13^***^	11.17	0.000
AL	23.49 ± 0.74	24.69 ± 1.09^***^	-6.439	0.000
Gender (n, %)			-0.606	0.546
Male	27(0.55)	26(0.49)		
Female	22(0.45)	27(0.51)		
Father’s vision status (n, %)			16.823	0.001
Emmetropia	28(57.1)	10(18.9) ^**^		
Low myopia	10(20.4)	15(28.3) ^**^		
Moderate myopia	8(16.3)	21(39.6) ^**^		
Severe myopia	3(6.1)	7(13.2) ^**^		
Mother’s vision status (n, %)			10.407	0.015
Emmetropia	22(44.9)	13(24.5) ^*^		
Low myopia	14(28.6)	13(24.5) ^*^		
Moderate myopia	9(18.4)	25(47.2) ^*^		
Severe myopia	4(8.2)	2(3.8) ^*^		
Daily use of electronic devices (n, %)			5.733	0.017
≤2h	43(87.8)	36(67.9) ^*^		
>2h	6(12.2)	17(32.1) ^*^		
Picky eating and selective eating behaviors (n, %)			4.559	0.033
Yes	15(30.6)	7(13.2) ^*^		
No	34(69.4)	46(86.8) ^*^		
Weekly protein intake (n, %)			0.315	0.575
High	47(95.9)	50(94.3)		
Low	2(4.1)	3(5.7)		
Habit of using electronic devices or books while in transit (n, %)			0.167	0.682
Yes	13(26.5)	16(30.2)		
No	36(73.5)	37(69.8)		
Daily outdoor activity duration			0.075	0.784
≤2h	39(79.6)	41(77.4)		
>2h	10(20.4)	12(22.6)		
Weekly intake of vegetables and fruits (n, %)			0.090	0.764
High	18(36.7)	21(39.6)		
Low	31(63.3)	32(60.4)		
Weekly intake of sweets (n, %)			2.734	0.098
High	35(71.4)	45(84.9)		
Low	14(28.6)	8(15.1)		
Weekly intake of barbecued and fried foods (n, %)			0.665	0.415
High	36(73.5)	35(66.0)		
Low	13(26.5)	18(34.0)		
Use of nutrients such as beta-carotene, lutein, etc. (n, %)			0.004	0.948
Yes	9(18.4)	10(18.9)		
No	40(81.6)	43(81.1)		

### 3.2 Gut microbial diversity analysis

We employed α diversity analysis to assess gut microbial community diversity. The observed and chao1 indices reflect species richness, while the Shannon and Simpson indices comprehensively evaluate both richness and evenness, serving as robust indicators of community diversity.

**Figure 1:**
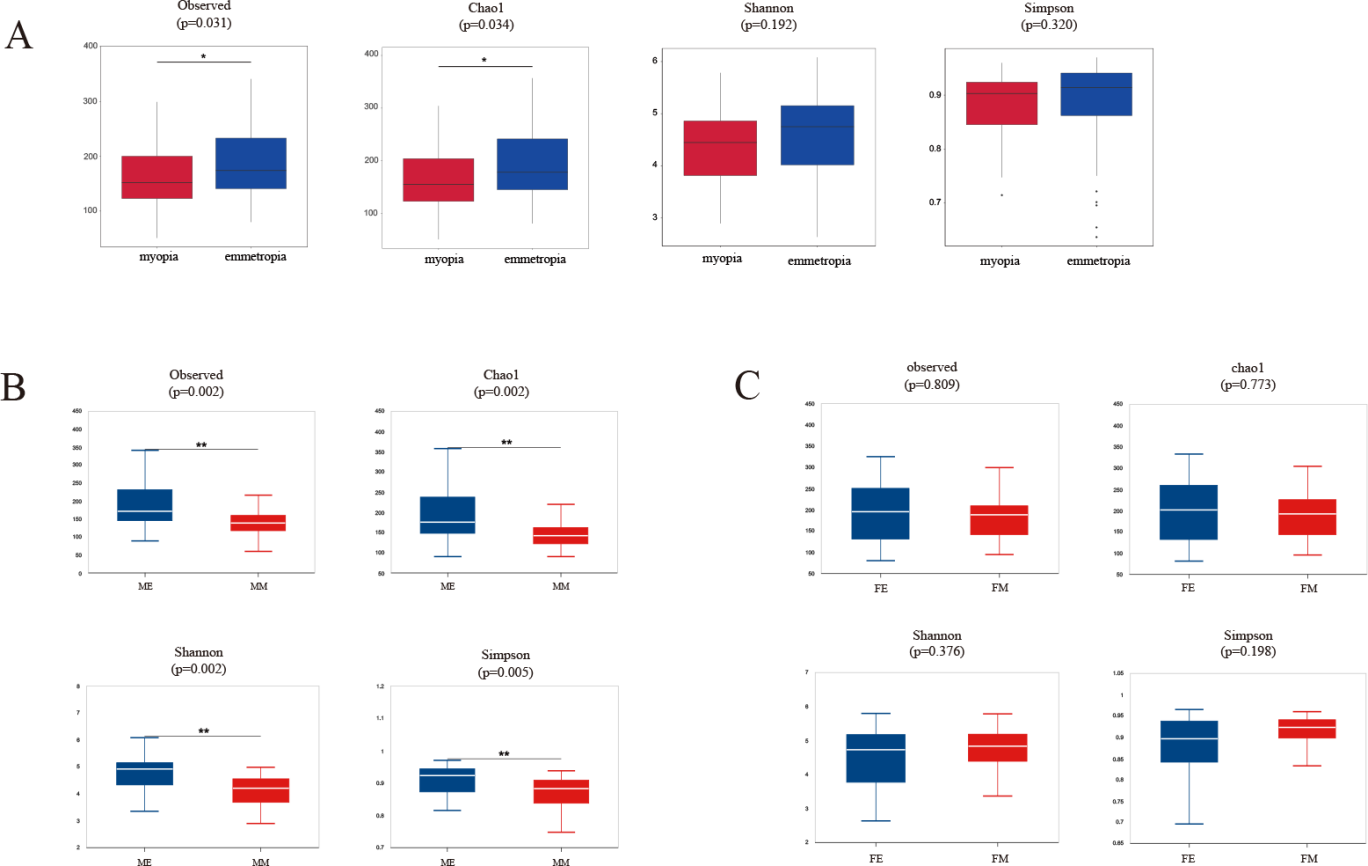
Box plot of α-diversity indices. **(A)** All adolescents; **(B)** Male subgroup; **(C)** Female subgroup. The Observed and Chao1 indices reflect species richness, while the Shannon and Simpson indices reflect diversity. *P<0.05, **P<0.01.

Wilcoxon signed-rank test results revealed significant intergroup differences in observed (P=0.031) and chao1 (P=0.034) indices between myopic and non-myopic adolescents, whereas Shannon and Simpson indices showed no significant differences (P>0.05) (**Figure 1A**). This suggests that myopia-related gut microbiota changes are mainly characterized by loss of specific taxa (reduced richness) rather than a marked alteration in overall evenness.

Notably, in the male adolescent group, all α-diversity metrics (observed, chao1, Shannon, and Simpson) showed extremely significant differences between myopic and non-myopic individuals (P < 0.01) (**Figure 1B**). In contrast, no significant differences were observed in any α-diversity metrics among female adolescents (P > 0.05) (**Figure 1C**). These findings suggest that differences in gut microbiota diversity between myopic and non-myopic adolescents are associated with sex, with significant intergroup diversity differences observed in male adolescents but not in females.

To assess microbial community complexity and compare intergroup differences, β-diversity analysis was performed using weighted UniFrac distance. Wilcoxon tests revealed no significant differences in β-diversity indices between myopic and non-myopic adolescents, either in the overall adolescent cohort or when analyzed by sex (P > 0.05).

**Figure 2:**
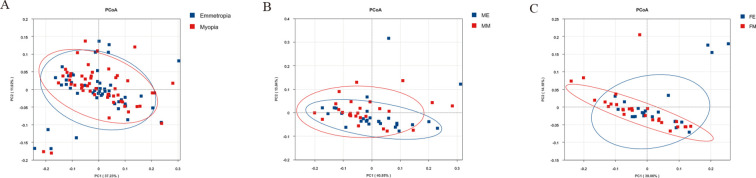
Principal coordinate analysis (PCoA) plot. **(A)** All adolescents; **(B)** Male subgroup; **(C)** Female subgroup. Each dot represents a sample, with different colors and shapes indicating groups: ME, male emmetropia; MM, male myopia; FE, female emmetropia; FM, female myopia. Percentages on axes represent the proportion of community structure variation explained by each principal coordinate.

Principal Coordinate Analysis (PCoA) extracts the most dominant elements and structures from multidimensional data through a series of eigenvectors and eigenvalues. This study performed PCoA based on weighted UniFrac distances, visualizing the most contributory principal coordinate combinations. Closer sample distances indicate greater similarity in species composition structures.The PCoA plot (**Figure 2**) revealed that both male and female adolescent samples, regardless of myopia status, clustered together, indicating highly similar microbial community structures.Consistent with this, no significant differences in beta-diversity indices were detected between groups (P > 0.05), suggesting that myopia is not associated with a global shift in gut microbial community architecture but rather with changes in specific taxa.”

### 3.3 Community composition and differential abundance

**Figure 3:**
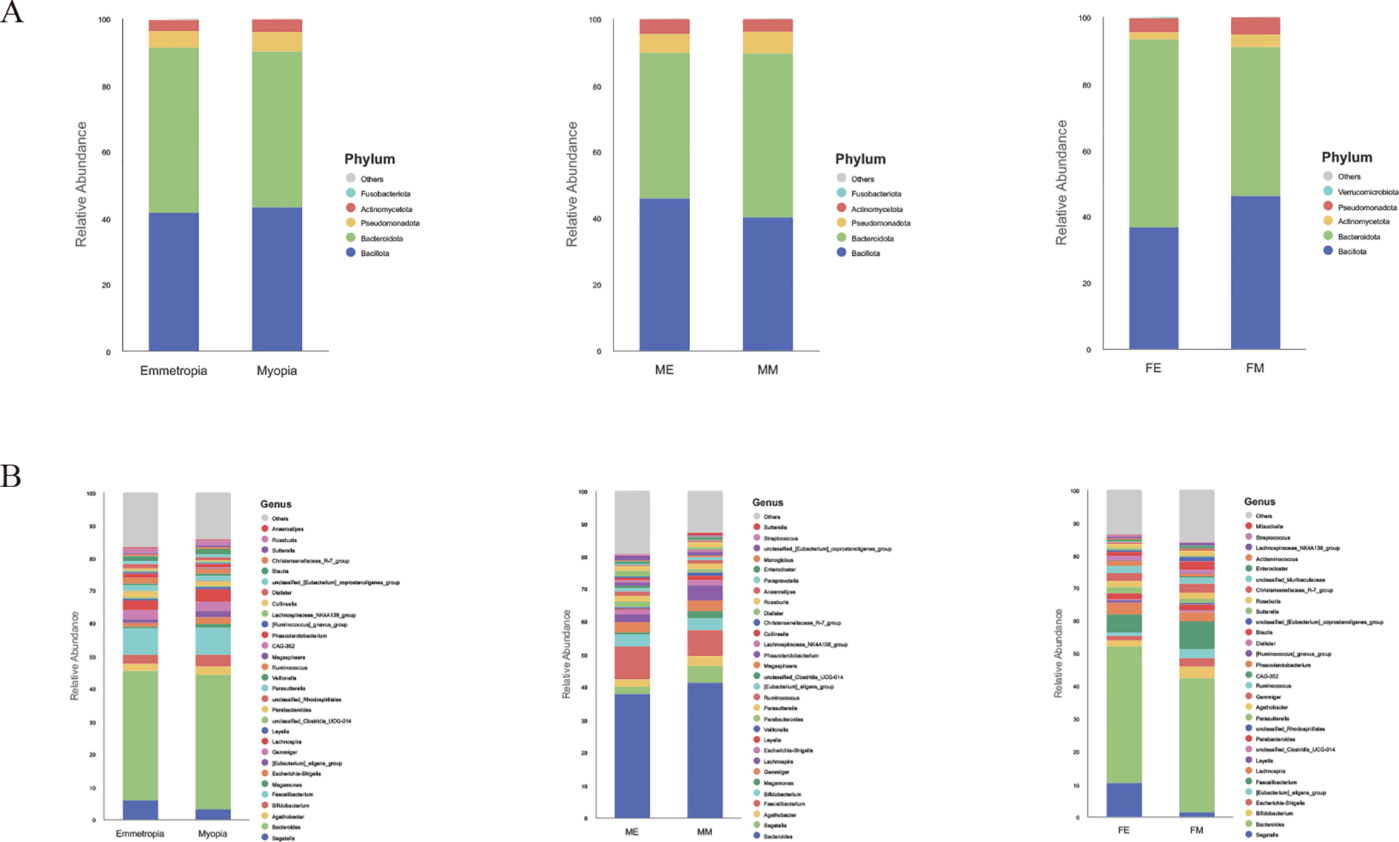
Bar plots of relative species abundance at the phylum and genus levels. **(A)** Relative abundance at the phylum level (top 4 dominant phyla: Bacteroidetota, Bacillota, Pseudomonadota, Actinomycetota); **(B)** Relative abundance at the genus level (top 30 dominant genera).

Based on species annotation results at different taxonomic levels, species with the top 5 highest relative abundances at the phylum level and the top 30 highest relative abundances at the genus level were selected for each group. Relative abundance bar charts were plotted (**Figure 3**). At the phylum level, the four most abundant phyla were Bacteroidetota, Bacillota, Pseudomonadota, and Actinomycetota. Metagenome sequencing analysis revealed that among male subjects, non-myopic individuals exhibited significantly higher Verrucomicrobiota phylum abundance than myopic individuals.

At the genus level, non-myopic subjects exhibited significantly higher abundance of the [Eubacterium]_ruminantium_group genus compared to myopic subjects. Among male subjects, non-myopic individuals showed significantly higher abundance of the unclassified_Clostridia_UCG-014, Alistipes, Oscillibacter, Akkermansia, unclassified_UCG-010, and Oscillospira genera than myopic males;while myopic individuals exhibited significantly lower abundances of Veillonella and Sutterella. Among female participants, non-myopic individuals showed significantly higher Paraprevotella abundance than myopic individuals; conversely, myopic individuals exhibited significantly higher Erysipelotrichaceae_UCG-003 abundance than non-myopic individuals.

**Figure 4:**
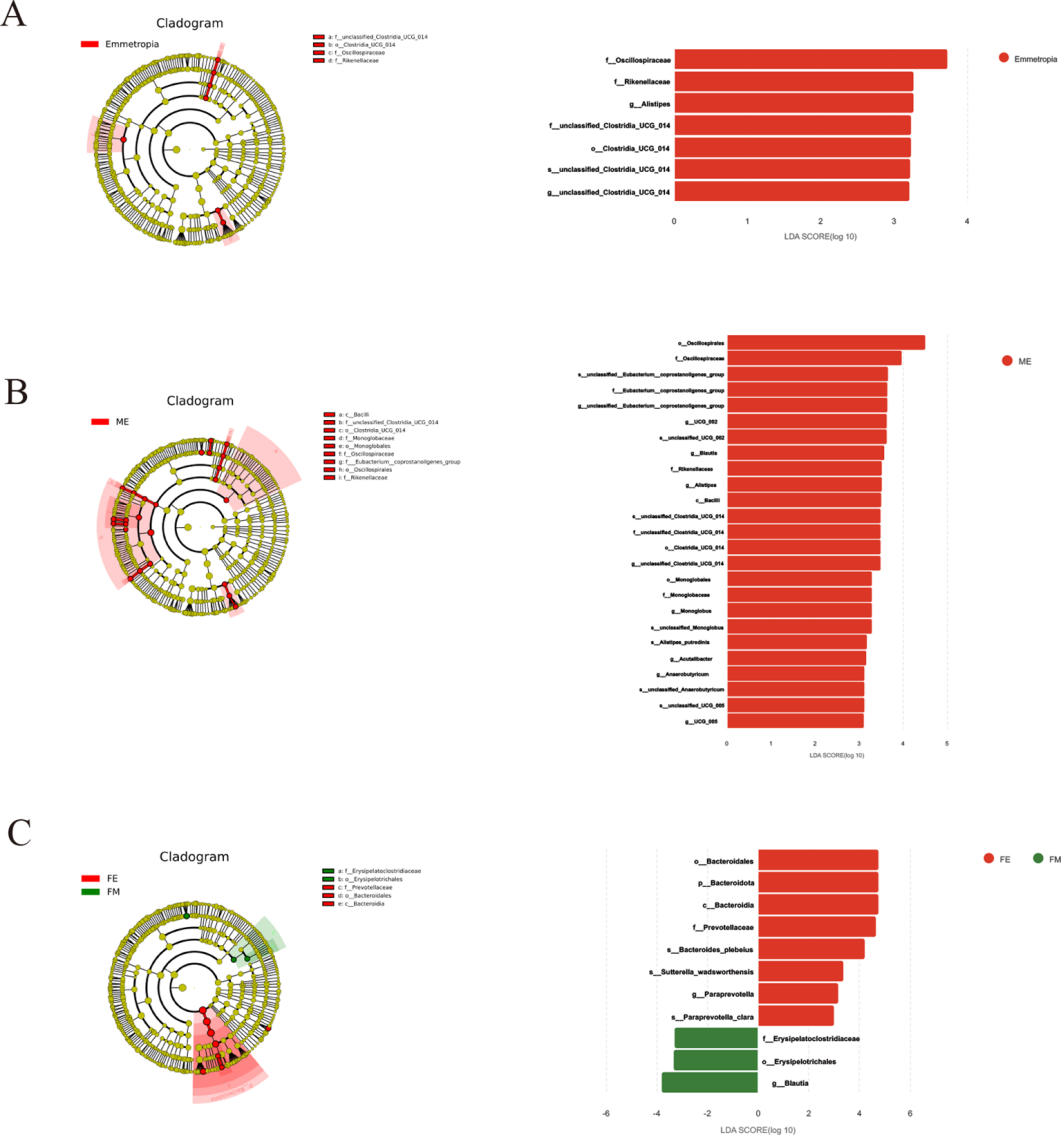
LEfSe cladogram(left) and bar chart(right). **(A)** All adolescents; **(B)** Male subgroup; **(C)** Female subgroup. In the left panel, concentric circles radiating outward represent taxonomic levels from phylum to genus; each small circle at a given level represents a taxon, with diameter proportional to relative abundance. In the right panel, bar length indicates the effect size (LDA score) of differentially abundant taxa; only taxa with LDA > 3 are shown.

LDA effect size analysis (LEfSe) was performed on the gut microbiota of non-myopic and myopic individuals, including evolutionary branch diagrams and bar charts (**Figure 4**). In the evolutionary branch diagram, concentric circles radiating outward represent taxonomic levels from phylum to genus. Each small circle at a taxonomic level denotes a taxon at that level, with circle diameter proportional to relative abundance. Bar chart length indicates the effect size of differential species, i.e., LDA values. Of note, differential abundance analysis by metagenomeSeq was performed without multiple comparison correction; the results are exploratory and may have an increased risk of false positives.

### 3.4 Functional pathway prediction

**Figure 5:**
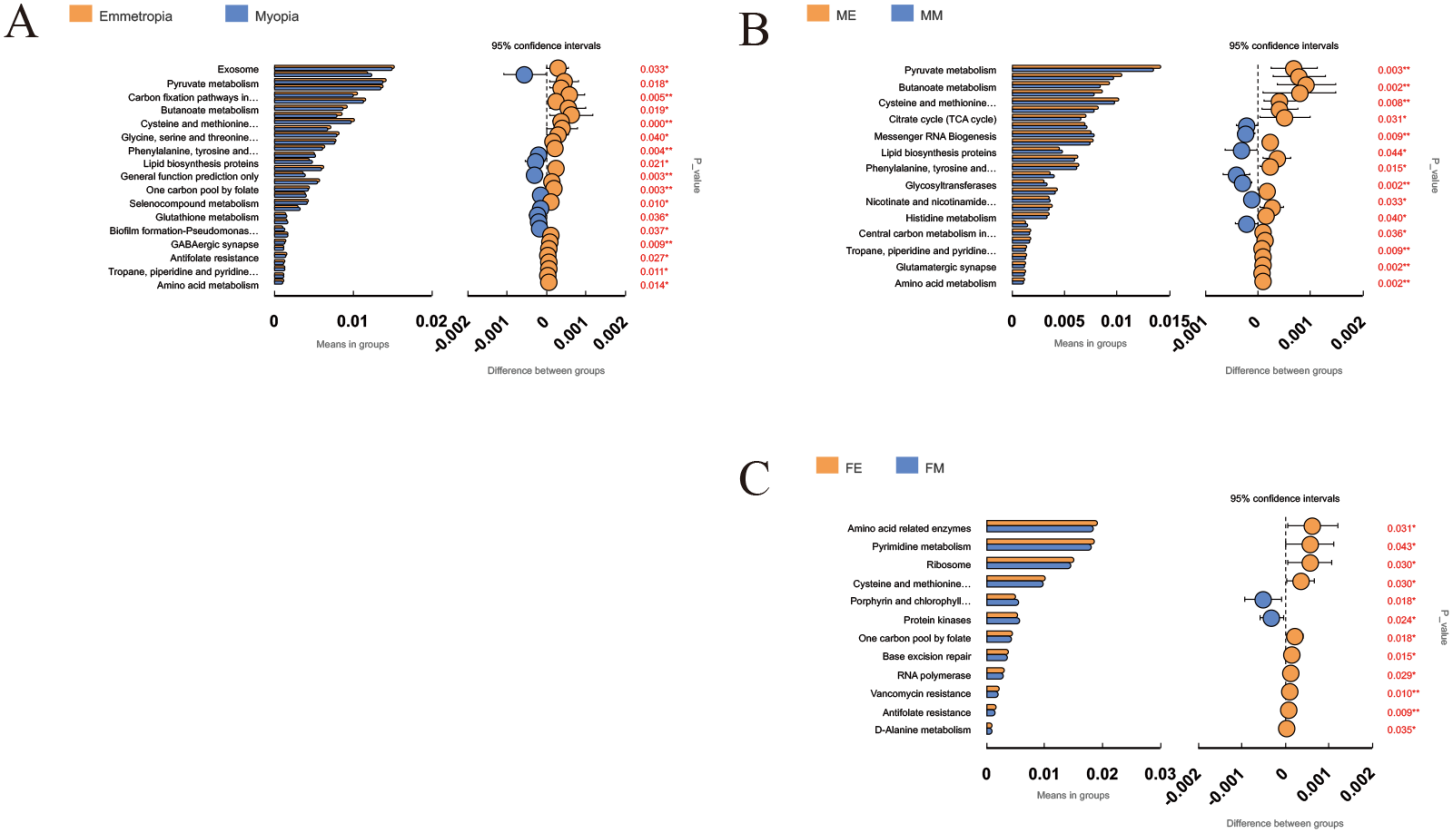
Tax4Fun functional pathway prediction. **(A)** All adolescents; **(B)** Male subgroup; **(C)** Female subgroup. Left panel: KEGG pathways (level 3) with significant differences between groups; bar height represents mean relative abundance. Right panel: 95% confidence intervals for the differential pathways; horizontal lines indicate effect size and credible range. Comparisons between groups were performed using Student’s t-test.

To explore functional pathways of gut microbiota associated with myopia, we employed the Tax4Fun tool. This tool aligns 16S rRNA sequences from gut microbiota with known functionally annotated prokaryotic genomes to infer potential functions of microbial communities. Differences between groups were tested using t-tests. **Figure 5** left panel displays differential functions between groups, with bars representing the mean differential functional abundance across different groups; the right panel shows the confidence level of intergroup differences.

### 3.5 Random forest prediction model

**Figure 6:**
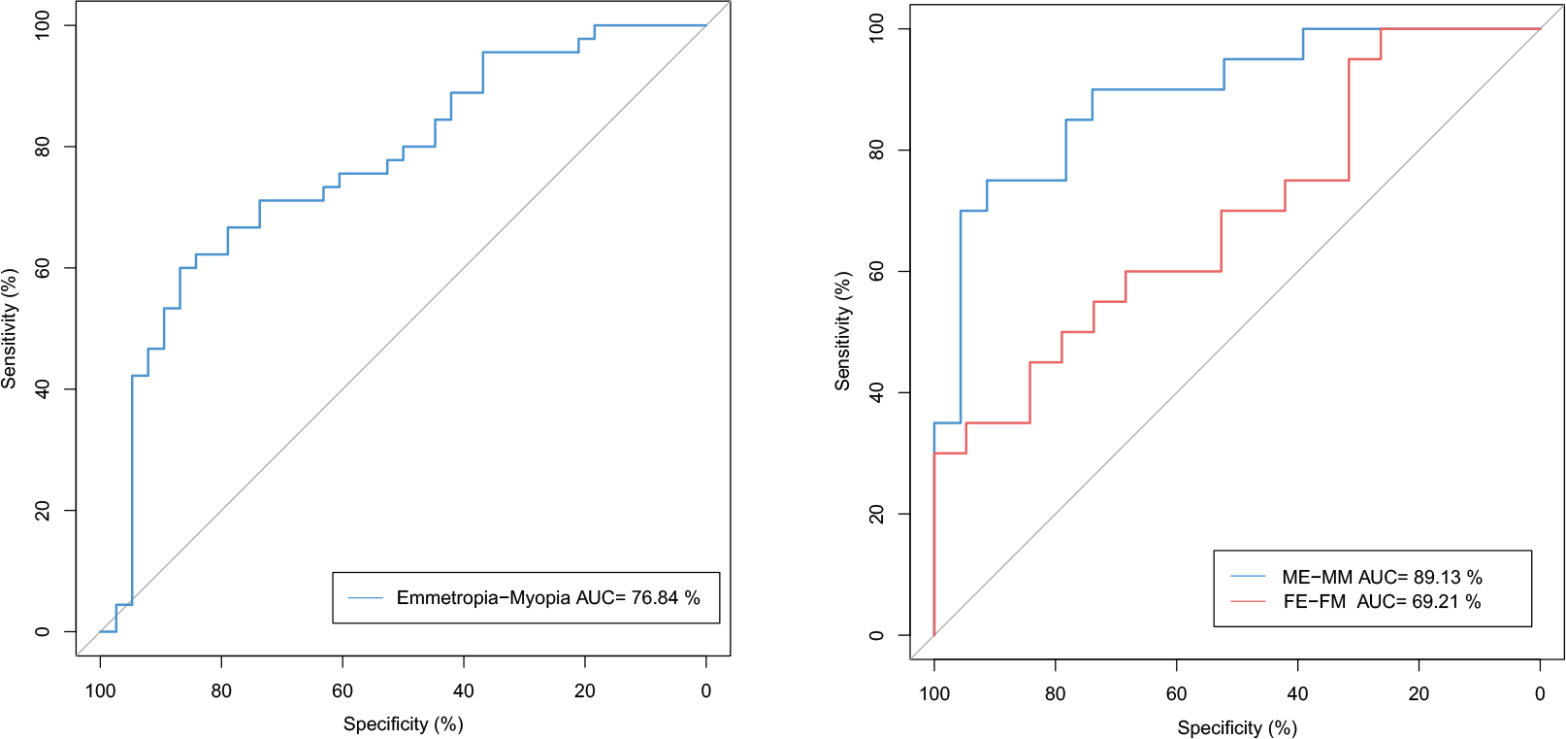
ROC curve of the random forest test set. X-axis: false positive rate (1 - specificity); Y-axis: true positive rate (sensitivity). Blue curve: overall model (AUC = 76.84%); green curve: male subgroup (AUC = 89.13%); orange curve: female subgroup (AUC = 69.21%).

Random forest is a classic machine learning model based on decision tree algorithms. We constructed a classification prediction model for myopia versus non-myopia using genus-level relative species abundance as features. Data were divided into training and test sets. The classification function was trained on the training set to optimize classification performance, and model performance was evaluated using receiver operating characteristic (ROC) curves (**Figure 6**). Our model demonstrated high classification accuracy on the test set (AUC = 76.84%). Gender-specific analysis revealed higher prediction accuracy for males (AUC = 89.13%) compared to females (AUC = 69.21%).

## 4 Discussion

The relationship between gut microbiota and myopia has been a subject of increasing interest in recent years, with emerging evidence suggesting that the gut microbiome may influence ocular health (Zysset-Burri et al., 2023; Labetoulle et al., 2024). Our study, based on 16S rRNA sequencing, aimed to explore the gut microbiota composition in myopic and non-myopic adolescents and investigate potential gender-specific differences. Interestingly, while no significant differences in overall gut microbial diversity or community structure were observed between myopic and non-myopic adolescents, a more nuanced analysis revealed that male myopic subjects exhibited significantly lower α-diversity compared to their non-myopic counterparts. This gender-specific difference suggests that sex may play a critical role in shaping the gut microbiota-myopia relationship, which warrants further investigation.

Emerging evidence suggests that gut dysbiosis may serve as a shared pathological link between myopia and other systemic or ocular comorbidities.Several studies have reported shared microbial features between myopia and metabolic comorbidities,inflammation and autoimmune diseases,indicating that gut dysbiosis may represent a common pathological substrate linking these conditions (Lv et al., 2024; Gu et al., 2025).

Reduced α-diversity of the gut microbiota has been associated with elevated inflammation levels and lipid metabolism abnormalities (Wilmanski et al., 2019), which play crucial roles in scleral remodeling and axial elongation.The observed lower α-diversity in males with myopia suggest that their gut microenvironment is more sensitive to myopia-related pathophysiological processes. Such sex-specific pattern aligns with known hormonal influences on microbial ecology, as androgens and estrogens differentially regulate bacterial composition and immune tone (Markle et al., 2013; Valeri and Endres, 2021). The differential regulation by sex hormones could play a role in shaping the microbial landscape in male subjects, making their gut microenvironment more responsive to such changes.

Although no significant differences in community structural diversity were found between myopic and non-myopic groups at the broader taxonomic level, a more detailed analysis of species-level differences via MetagenomeSeq analysis revealed statistically significant variations in the abundance of Verrucomicrobiota phylum at the phylum level between male myopic and non-myopic individuals. Specifically, in the male cohort, the non-myopic group exhibited an enrichment of several genera with potentially beneficial metabolic functions, including Akkermansia (a member of the Verrucomicrobiota phylum), Alistipes, Oscillibacter, and unclassified_Clostridia_UCG-014.

Akkermansia, known for its role in enhancing intestinal mucosal barrier function and regulating both immunity and metabolism, is often found in reduced abundance in individuals with various metabolic disorders (Ioannou et al., 2025). Similarly, Alistipes is recognized for its ability to alleviate intestinal inflammation and modulate immune functions, and it has been linked to a reduced risk of acute anterior uveitis, although its abundance is elevated in patients with diabetic retinopathy (Mi et al., 2025; Adhikary et al., 2026). Oscillibacter is involved in butyrate production, a short-chain fatty acid that has been shown to protect retinal neurovascular units from damage (Gong et al., 2025). Notably, Mendelian randomization analysis suggests that increased abundance of Oscillospira genus maybe a risk factor for allergic conjunctivitis (Liu et al., 2023).

In Contrast, certain genera such as Veillonella and Sutterella, which were enriched in myopic males, have been linked to inflammatory conditions (Kaakoush, 2020; Schirmer et al., 2024). These findings indicate the gut microbiota of myopic males may exhibit mild pro-inflammatory characteristics compared to their non-myopic counterparts. Fewer bacterial genera were differentially abundant between myopic and non-myopic females. However, the non-myopic female group exhibited enrichment in Paraprevotella, a genus known for its ability to degrade trypsin, protect intestinal immunoglobulin IgA, and suppresses enterovirus infection (Li et al., 2022). These distribution patterns suggest that the microbiota composition in non-myopic males may offer greater protection in terms of metabolic regulation and immune modulation, while the gut microbiota of myopic males may favor a more pro-inflammatory environment.

Previous literature has debated the predictive efficacy of gut microbiota for myopia. In this study, we constructed a random forest model to evaluate the predictive value of gut microbiota differences have predictive value for myopia, with higher predictive efficacy observed in males. This suggests that the male gut microbiome may have more pronounced and specific associations with myopia.It should be emphasized that refraction testing is far simpler, more cost-effective, and more accurate; therefore, gut microbiota testing lacks clinical value as a diagnostic substitute. The predictive model developed in this study is intended solely for exploring the underlying mechanisms.

However, several limitations of this study must be considered. First, due to the cross-sectional design, all findings should be interpreted as associations only, not as evidence of causation.The observed association between picky eating and non-myopic status (**Table 1**) raises the possibility that dietary habits may influence gut microbiota composition, but we cannot determine the causal direction among diet, gut microbiota, and myopia. Second, although we identified several genera that differed between myopic and non-myopic adolescents, we did not perform culture experiments or functional validation of these differential microbiota. Such experiments are essential to confirm their biological relevance. Future studies should isolate and culture the identified candidate bacteria (e.g., Akkermansia, Alistipes, Oscillibacter) and conduct animal experiments or fecal microbiota transplantation to test their causal roles in myopia development. Third, the single-center sample size, particularly after gender-specific stratification, was relatively small, which may have introduced selection bias. Future studies should aim to expand the sample size and establish more refined subgroups based on factors such as myopia severity, progression rate, and presence of retinal pathology. Such stratification will help to further elucidate the complex relationship between gut microbiota and various phenotypes of myopia.Fourth, while we identified parental refractive status, electronic device use, and picky eating as factors associated with myopia in our cohort, these variables were not entered as covariates in a multivariate model to assess their independent or interactive effects on the gut microbiota–myopia relationship. Given the exploratory nature of this study and the limited sample size after sex stratification, we prioritized sex-stratified analysis to control for sex as a major biological variable. Future studies with larger sample sizes and multivariable adjustment are warranted to disentangle the contributions of these potential confounders.

Moreover, this study did not explore the underlying mechanisms through which gut microbiota may influence myopia development.Animal experiments should be incorporated in future research to validate molecular mechanisms underlying the gut-eye axis and its modulation by sex hormones.

## Data Availability

The data presented in the study are deposited in the NCBI SRA repository, accession number PRJNA1463993
